# Probiotic Supplements Improve Blood Glucose and Insulin Resistance/Sensitivity among Healthy and GDM Pregnant Women: A Systematic Review and Meta-Analysis of Randomized Controlled Trials

**DOI:** 10.1155/2021/9830200

**Published:** 2021-09-22

**Authors:** Yu-Qing Pan, Qing-Xiang Zheng, Xiu-Min Jiang, Xiao-Qian Chen, Xiao-Yun Zhang, Jing-Ling Wu

**Affiliations:** ^1^School of Nursing, Fujian Medical University, No. 1 Xuefu North Road, University Town, Shangjie Zhen, Minhou County, Fuzhou City, Fujian Province, China; ^2^Fujian Maternity and Child Health Hospital Affiliated to Fujian Medical University, No. 18 Daoshan Street, Gulou District, Fuzhou City, Fujian Province, China; ^3^Fujian Obstetrics and Gynecology Hospital Affiliated to Fujian Medical University, No. 18 Daoshan Street, Gulou District, Fuzhou City, Fujian Province, China; ^4^School of Nursing, Fujian University of Traditional Chinese Medicine, Fuzhou, China

## Abstract

**Background:**

Probiotic supplements may be seen as a promising way to improve glucose metabolism. This study aimed to evaluate the effects of probiotic supplements on blood glucose, insulin resistance/sensitivity, and prevention of gestational diabetes mellitus (GDM) among pregnant women.

**Methods:**

Eleven electronic databases were searched from inception to May 2020. Two authors independently identified randomized controlled trials (RCTs), assessed the eligibility and quality of the included studies, and then extracted data. The primary outcomes were fasting plasma glucose (FPG), 1 h and 2 h plasma glucose after 75 g oral glucose tolerance test (OGTT), HbA1c, fasting plasma insulin, insulin resistance, and insulin sensitivity. Fixed and random effect models were used to pool the results.

**Results:**

A total of 20 RCTs involving 2972 participants were included according to the inclusion and exclusion criteria. The pooled results of this research showed that probiotic supplements could reduce the level of FPG (mean difference (MD) = −0.11; 95% CI = −0.15 to −0.04; *P*=0.0007), serum insulin (MD = −1.68; 95% CI = −2.44 to −0.92; *P* < 0.00001), insulin resistance (MD = −0.36; 95% CI = −0.53 to −0.20; *P* < 0.00001), and insulin sensitivity (MD = −21.80; 95% CI = −31.92 to −11.67; *P* < 0.00001). Regarding the subgroup analysis of different pregnant women, the effects of probiotics on FPG, insulin, and insulin resistance were more obvious among GDM and healthy women than among overweight/obese women. Furthermore, the differences were not significant in HbA1c (MD = −0.05; 95% CI = −0.12 to 0.03; *P*=0.23), 1 h OGTT (MD = −0.07; 95% CI = −0.25 to 0.10; *P*=0.42), and 2 h OGTT (MD = −0.03; 95% CI = −0.17 to 0.12; *P*=0.72).

**Conclusion:**

This review found that probiotic supplements had certain functions to reduce the level of FPG and improve insulin, insulin resistance, and insulin sensitivity, especially for GDM and healthy pregnant women.

## 1. Introduction

Gestational diabetes mellitus (GDM) is the most common pregnancy complication, and its prevalence is continually rising worldwide due to increased obesity and the average age of pregnant women [1], from 4.5% to 20.3% in the Western Pacific [2] and 14.8% in China [3]. Overweight and obesity can contribute to half of GDM's prevalence [4]. Meanwhile, obesity occurs in up to 30% of women [5], and obese women have a higher risk for GDM in comparison with normal-weight women [6]. Both GDM and obesity induce metabolic traits, including hyperglycemia, hyperinsulinemia, and insulin resistance [7, 8], as well as imposing a huge economic burden [1, 9, 10], especially in developing countries. GDM and obesity also cause ongoing maternal and neonatal health problems [2, 7, 8, 11–13]. Women with GDM are more likely to develop diabetes at rates of 20%–60% in five to ten years after pregnancy, and the incidence of metabolic diseases in their offspring also significantly increases [13]. Therefore, the prevention of obesity may be directly associated with a lower risk of GDM, and the prevention and treatment of hyperglycemia and insulin resistance among pregnant women have become a global concern.

Lifestyle intervention is the major method to control maternal hyperglycemia and insulin resistance, including medical nutrition therapy, exercise intervention, and self-monitoring of blood glucose [14]. Women with GDM whose blood sugar cannot be controlled at an ideal level by diet or exercise should be accepted for pharmacological therapy [14]. Although insulin therapy is the most common and safest pharmacological therapy, it is very labor-intensive and time-consuming for nurses and creates a financial burden on women with GDM [9]. Oral medications, such as metformin and sulfonylureas, are associated with higher risks for adverse pregnancy outcomes, such as large-for-gestational age, neonatal hypoglycemia, and birth injury. Pregnant women may also face many barriers during clinical implementation [15–17]. Owing to the poor management of lifestyle intervention and the limitations of pharmacotherapy, seeking a better way to improve hyperglycemia and insulin resistance is essential.

Probiotic is defined as beneficial live microorganisms in the host when they reach an adequate dose [18], and it plays an important role in improving the intestinal microenvironment, modulating the immune system, and preventing systemic disease and inflammation [19]. Previous studies have demonstrated that gut microbiota promotes the digestion of complex polysaccharides to produce monosaccharides and short-chain fatty acids (SCFAs) [20], which have positive relations with metabolism [21]. The composition and function of obese-diabetic microbiota change in comparison with healthy gut microbiota, which leads to metabolic disorders such as overweight/obesity, elevated blood glucose, insulin resistance, and inflammation [22, 23]. Accordingly, probiotics may be seen as a potential, economic, and practical approach to improve blood glucose and insulin resistance/sensitivity among pregnant women.

Even though previous studies indicated the effects of probiotics in preventing and treating GDM, some conflicting conclusions were still drawn from other studies. Zhang et al. (2019) [24] showed that probiotics could reduce the blood glucose level of women with GDM. On the contrary, several system reviews indicated that probiotic supplements did not reduce the blood glucose of women with GDM in comparison with the placebo group [25–27]. Moreover, in another two system reviews [28, 29], such probiotic supplements had a positive effect on women without a GDM diagnosis. Less is also known about the effects of probiotics on different pregnancy status as well as the duration, dosages, and type of probiotic interventions.

This study aimed to synthesize more high-quality randomized controlled trials (RCTs) to provide evidence of the effects of probiotic supplements on glycemic control, insulin resistance/sensitivity, and prevention of GDM among pregnant women.

## 2. Method

This review completely followed the PRISMA guidelines [30].

### 2.1. Search Strategy

We searched 11 electronic databases (Embase, Web of Science, Medline, PubMed, Cochrane, CINAHL, Clinicaltrials.gov, China National Knowledge Infrastructure, WanFang Data, Chinese Scientific Journal Database, and SinoMed) from April 2020 to May 2020. The search strategy combined MeSH terms and free words that were listed as follows: “pregnant,” “obstetric,” “obesity,” “overweight,” “gestational diabetes,” “gestational diabetes mellitus,” “probiotic,” “symbiotic,” “lactobacilli,” “streptococc,” “bifidobacter,” “saccharomy,” “yeast,” “yogurt,” and “bacteria.” The retrieval was adjusted to different features of each database. To expand the retrieval area and advance the recall of the search engine, the reference list of included studies and relevant reviews were further tracked through the snowballing method.

### 2.2. Selection Criteria

The inclusion criteria of this study were as follows: (1) RCTs; (2) studies published in Chinese or English; (3) pregnant women with or without overweight or obesity (body mass index (BMI) 25.0–29.9 kg/m2 or ≥30.0 kg/m2 at the first antenatal visit, resp.), GDM (diagnosed in the second or third trimester of pregnancy, without identified diabetes before gestation), and over 16 years old; and (4) probiotic supplement was used as an intervention method.

Studies were excluded if they constituted duplicated publication, the information of glucose index (e.g., fasting glucose, insulin, and HOMA-IR) was not reported, or the relevant data could not be extracted.

### 2.3. Data Extraction

Two review authors (Pan and Zheng) independently selected literature according to the inclusion and exclusion criteria after screening the title, abstract, and full text and then extracted data. The information we extracted from the eligible studies included (1) basic information of the study: first author, year of publication, country, and sample size; (2) characteristics of participants: age and BMI at baseline; (3) intervention details: probiotic species, dose, frequency, and duration; (4) primary outcomes, including fasting plasma glucose (FPG), 1 h and 2 h plasma glucose post 75 g oral glucose tolerance test (OGTT), and glycated Hb (HbA1c); and (5) secondary outcomes, including fasting plasma insulin (FPI), homeostasis model assessment insulin resistance (HOMA-IR), homeostasis model of assessment-estimated *β* cell function (HOMA-B), quantitative insulin sensitivity check index (QUICKI), and incidence of GDM. We resolved any disagreement through discussion or turned to a third reviewer (Jiang) when consensus was not reached.

### 2.4. Quality Assessment

Two review authors (Pan and Zheng) used the Cochrane risk of bias tool, Review Manager 5.3, to evaluate independently the methodological quality of each RCT. The classification of risk bias included (1) random sequence generation, (2) allocation concealment, (3) blinding of participants and personnel, (4) blinding of outcomes assessment, (5) incomplete outcome data, (6) selective reporting, and (7) other biases. Each classification was rated as “high risk,” “low risk,” or “unclear.” Once more than one entry was assessed as “high risk,” the quality of study would be regarded as high risk for bias. Any disagreement was resolved through discussion or by consulting a third reviewer (Jiang).

### 2.5. Data Synthesis

Review Manager software (version 5.3) was used for meta-analysis following the Cochrane handbook [31]. Two review authors (Pan and Zheng) cross-checked the entered data to ensure that they were strictly correct. Continuous variables were calculated with the mean difference (MD). Statistical heterogeneity was assessed with the Chi^2^ and *I*^2^. If *P* < 0.01 for the Chi^2^ test and *I*^2^ < 50% indicated that heterogeneity was not significant, then the fixed-effects model was utilized to merge the results. Otherwise, the meta-analysis used the random-effects model (*P* > 0.01for the Chi^2^ ≥ test and *I*^2^ > 50%). Moreover, to explore the potential clinical heterogeneity, a subgroup analysis was performed for each group meta-analysis according to different participants, types, dosages, and duration of probiotic intervention. If *I*^2^ was still greater than 50%, then the sensitivity analysis was conducted to guarantee the stability of results and remove the dubious studies. The descriptive analysis method was adopted if substantial heterogeneity still existed. The 95% confidence intervals (95% CI) were calculated in each statistical analysis, and *P* < 0.05 was regarded as significant for analysis. A funnel plot test was utilized to assess potential publication bias if more than 10 studies were included.

## 3. Results

**Figure 1:**
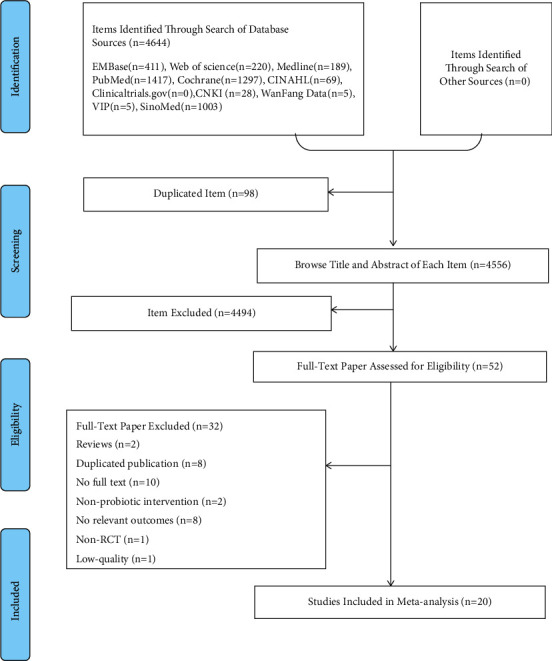
PRISMA flowchart of study selection.

A total of 4,644 articles were identified by searching 11 electronic databases. Fifty-two studies were kept after irrelevant articles (*n* = 4474), duplication (*n* = 98), and reviews (*n* = 20) were removed through screening titles and abstracts. Of the remaining studies retrieved, ten studies were protocols, two studies were reviews, and eight studies were duplicated publications. Among the rest of the studies, two studies did not utilize probiotics to intervene, eight studies reported irrelevant outcomes, and one study was a case report. The lone Chinese literature just reported the incident of GDM without any other relevant index. Hence, we finally included 20 RCTs based on a careful checking of the full text according to the selection criteria (Figure 1).

### 3.1. Characteristics of Included Studies

Twenty studies [32–51] with a total of 2,972 participants were included (Table 1). Each study had an average of 148 participants, ranging from 50 to 433, from New Zealand, Australia, Iran, Ireland, Finland, and Thailand. The participants were divided into three subgroups: overweight or obese pregnant women, women with GDM, and healthy pregnant women. The intervention types included probiotic capsules (16 studies [32–34, 36–45, 48, 51]), food (one study [47]), and probiotic yogurt (three studies [35, 46, 50]). Different species and combinations of probiotics were used in each study. The frequency of intervention in most studies was once per day. The duration of intervention in included studies was four weeks, six weeks, eight weeks, nine weeks, and from enrolment until delivery. According to the measurement time of each outcome indicator, this review divided the duration into two subgroups: short term (≤12 weeks) and long term (>12 weeks). The most common species of probiotics included *Lactobacillus rhamnosus*, *Bifidobacterium lactis*, *Lactobacillus acidophilus*, *Lactobacillus casei*, and *Lactobacillus fermentum*. The doses of probiotics ranged from 10^6^ colony-forming units (CFUs) to 10^11^ CFUs, and the most common dose was 10^9^ CFUs. The subgroups were set to small dose (<10^9^ CFU) [33–35, 39, 46, 47, 50] and large dose (≥10^9^ CFU) [32, 36–38, 40–45, 48, 49, 51]. Table 1 shows the characteristics of all included studies.

### 3.2. Quality Assessments of the Literature

**Figure 2:**
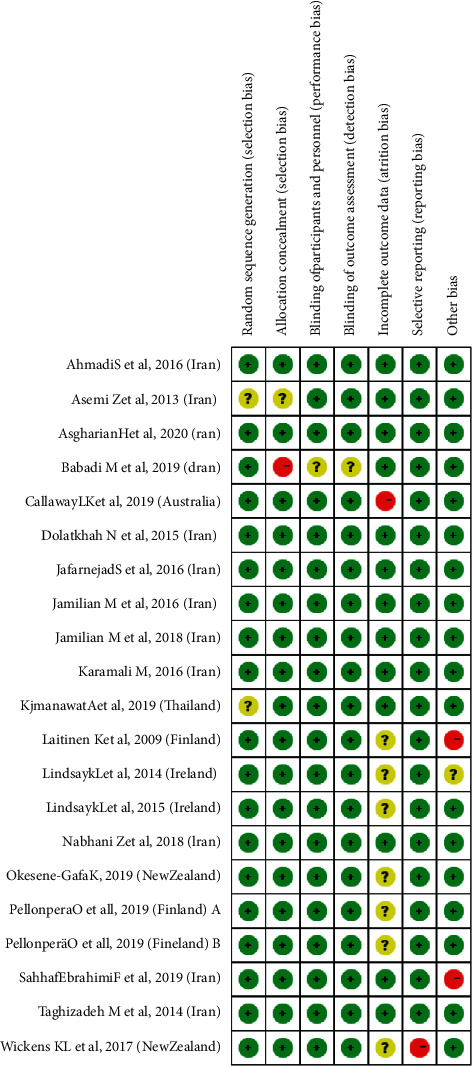
Assessments of risk of bias for included studies.

Figure 2 shows the methodological quality and risk for the bias of all included studies. Random sequence generation and allocation concealment were conducted in 18 included studies. Blinding of participants and personnel was reported in 19 studies, of which 5 studies reported triple blinding. A total of 7 studies used intention-to-treat analysis, 8 studies were considered “low risk for bias,” and only one study was evaluated as “high risk” for selective reporting. There was only one study (5%) with a small sample size, which was less than or equal to 50 participants.

### 3.3. Effect of Probiotic Supplements on Blood Sugar

#### 3.3.1. Assessment of Efficiency on FPG

In 20 studies with 2,555 participants, the FPG level was reduced in the intervention group (MD = −0.11; 95% CI = −0.15 to −0.04; *P*=0.0007) compared with that in the control group (Table 2). For the effects of probiotic supplementation on different participants, the pooled results indicated that the intervention group significantly differed from the placebo group among women with GDM (MD = −0.12; 95% CI = −0.24 to −0.01; *P*=0.04) and healthy pregnant women (MD = −0.07; 95% CI = −0.11 to −0.02; *P*=0.004). However, there were no significant differences in overweight/obese women for the FPG level. After sensitivity analyses, more concentrated results were obtained in the overweight/obese subgroup, changing from (MD = −0.09; 95% CI = −0.20 to 0.02; *P*=0.12) to (MD = −0.13; 95% CI = −0.21 to −0.06; *P*=0.0006), and the 95%CI (−0.24 to −0.01) was shortened (−0.32 to −0.13) in the subgroup of GDM. When the different duration of probiotic intervention was considered, all the results illustrated that short-term (MD = −0.13; 95% CI = −0.22 to −0.04; *P*=0.006) intervention benefitted pregnant women to improve the FPG level, whereas long-term intervention had no benefits. After sensitivity analysis, both short-term (MD = −0.12; 95% CI = −0.20 to −0.03; *P*=0.006) and long-term (MD = −0.11; 95% CI = −0.18 to −0.05; *P*=0.0005) interventions were effective and were only slightly different. In terms of the intervention types, probiotic capsule (MD = −0.008; 95% CI = −0.17 to −0.03; *P*=0.008) improved the FPG level more than probiotic yogurt did (MD = −0.09; 95% CI = −0.18 to 0.00; *P*=0.05), which was merely at the junction with zero boundaries. Only one study [47] reported probiotics in the form of diet. Significant heterogeneity (*I*^2^ = 77%) among the probiotic capsule subgroup was decreased after three skeptical studies were removed, and the result of the meta-analysis also improved precision. A large dose of probiotic supplement was statistically significant (MD = −0.10; 95% CI = −0.17 to −0.03; *P*=0.004).

#### 3.3.2. Assessment of Efficiency on 1 h and 2 h OGTT and HbA1C

Six included studies reported the OGTT, including 1,434 participants of 1 h OGTT and 1,491 participants of 2 h OGTT (Table 2). The results of the meta-analysis indicated that 1 h OGTT (MD = -0.07; 95% CI = −0.25 to 0.10; *P*=0.42) and 2 h OGTT (MD = −0.03; 95% CI = −0.17 to 0.12; *P*=0.72) did not have a significant difference between the intervention and control groups. In the subgroup analyses stratified by pregnancy status and intervention form, the results of 1 h OGTT (MD = −0.04; 95% CI = −0.24 to 0.16; *P*=0.71) and 2 h OGTT (MD = 0.01; 95% CI = −0.15 to 0.18; *P*=0.89) among overweight/obese pregnant women did not differ from those among the control group; probiotic capsule did not have a significant reduction in 1 h OGTT (MD = −0.23; 95% CI = −0.24 to 0.70; *P*=0.75) and 2 h OGTT (MD = 0.03; 95% CI = −0.11 to 0.18; *P*=0.65). Only one study reported OGTT in the subgroup of healthy women and probiotic yogurt. Neither of the above two subgroups drew statistical differences between the intervention and control groups. In the outcomes of 1 h and 2 h OGTT, a subgroup analysis of dosage could not be performed because of all included RCTs that adopted a large dose of probiotic bacteria. Meanwhile, HbA1c was reported in a total of 389 pregnant women in three studies. The results showed no significant difference (MD = −0.05; 95% CI = −0.12 to 0.03; *P*=0.23) between the intervention and control groups. No heterogeneity existed (*I*^2^ = 0%), and there were no available studies to conduct a subgroup analysis.

### 3.4. Effect of Probiotics on Insulin

#### 3.4.1. Assessment of Efficiency on Fasting Plasma Insulin

Information on the FPI level was measured in 16 studies involving 1,446 participants (Table 2). A significant reduction was observed in comparison with the intervention group and the control group (MD = −1.68; 95% CI = −2.44 to −0.92; *P* < 0.00001). Compared with the pregnancy status of overweight/obese, the level of FPI among women with GDM (MD = −2.40; 95% CI = −3.70 to −1.09; *P*=0.0003) and healthy women (MD = −1.77; 95% CI = −2.40 to −1.14; *P* < 0.00001) was significantly decreased. After sensitivity analysis, the results were more precise and *I*^2^ values were reduced to zero. For different probiotic intervention duration, short-term intervention could promote insulin control (MD = −1.55; 95% CI = −2.17 to −0.93; *P* < 0.00001) instead of long-term intervention (MD = 0.42; 95% CI = −2.38 to 3.18; *P*=0.77). After sensitivity analysis, the results of the short-term intervention were stable, but long-term intervention (MD = −1.32; 95% CI = −2.02 to −0.63; *P*=0.0002) had a positive effect on reducing the level of insulin. In terms of different intervention forms, the probiotic capsule might be effective in reducing insulin (MD = −1.66; 95% CI = −2.59 to −0.73; *P* < 0.00001). In this meta-analysis, both the small dose (MD = −0.29; 95% CI = −0.41 to −0.17; *P* < 0.00001) and large dose (MD = −0.29; 95% CI = −0.34 to −0.24; *P* < 0.00001) of probiotics had significant differences. Nevertheless, the heterogeneity decreased from 76% to 38%, and the pooled result was still stable after sensitivity analysis was performed.

#### 3.4.2. Assessment of Efficiency on HOMA-IR and HOMA-B

There were 16 studies involving 1,383 participants, where the measured HOMA-IR was significantly improved (MD = −0.36; 95% CI = −0.53 to −0.20; *P* < 0.00001) in comparison with the control group (Table 2). For different pregnancy status, the results illustrated that probiotic supplements in women with GDM (MD = −0.59; 95% CI = −0.88 to −0.30; *P* < 0.00001) and healthy women (MD = −0.34; 95% CI = −0.48 to −0.20; *P* < 0.00001) had a positive effect on HOMA-IR. However, positive results could not be obtained in overweight/obese women regardless of whether a sensitivity analysis was conducted or not. With regard to the different duration of interventions, the results of subgroup analyses showed that short-term intervention (MD = −0.47; 95% CI = −0.64 to −0.31; *P* < 0.00001) reaped benefits; long-term intervention had no positive result (MD = −0.15; 95% CI = −0.61 to 0.32; *P*=0.53), but the result of such intervention changed after sensitivity analysis (MD = −0.27; 95% CI = −0.47 to −0.08; *P*=0.006). Moreover, four studies with 242 participants measured HOMA-B (Table 2). Similarly, the results of the meta-analysis indicated that probiotics had positive effects on reducing the HOMA-B (MD = −21.80; 95% CI = −31.92 to −11.67; *P* < 0.00001). When it came to different pregnancy status, the pooled results showed that probiotics were also beneficial to women with GDM (MD = −25.25; 95% CI = −39.04 to −11.47; *P*=0.0003) and healthy women (MD = −17.74; 95% CI = −32.66 to −2.82; *P*=0.02). For the probiotic capsule subgroup, the pooled effectiveness on HOMA-IR (MD = −0.40; 95% CI = −0.63 to −0.18; *P*=0.0004) and HOMA-B (MD = −22.76; 95% CI = −32.32 to −12.19; *P* < 0.00001) was shown to have significant differences. Still, the dosage of probiotics did not affect the effect on HOMA-IR and HOMA-B.

#### 3.4.3. Assessment of Efficiency on QUICKI

Nine studies enrolling 582 participants measured QUICKI (Table 2). QUICKI was improved in the probiotic group in comparison with the control group (MD = 0.01; 95% CI = 0.00 to 0.01; *P*=0.001). For different pregnancy status, the results of this meta-analysis indicated that QUICKI in women with GDM (MD = 0.00; 95% CI = 0.00 to 0.01; *P*=0.03) and healthy women (MD = 0.02; 95% CI = 0.01 to 0.03; *P*=0.001) had a slight difference compared with women in the control group. Regarding the different types of probiotics, significant differences were shown in the probiotic capsule (MD = 0.01; 95% CI = 0.00 to 0.01; *P*=0.002). Given the number of studies, meta-analysis was conducted only for the large-dose group, and a statistical difference was observed between intervention groups and placebo groups.

### 3.5. Effect of Probiotics on the Incidence of GDM

The incidence of GDM was measured in seven studies with a total of 1,645 participants. There was no statistical difference between the intervention and control groups (RR = 1.03; 95% CI = 0.94 to 1.18; *P*=0.67). For different pregnant women, the same result was obtained (RR = 1.09; 95% CI = 0.95 to 1.25; *P*=0.24). Regarding the different duration and intervention types of probiotics, long-term intervention (RR = 1.03; 95% CI = 0.90 to 1.18; *P*=0.69) and probiotic capsule (RR = 1.05; 95% CI = 0.92 to 1.21; *P*=0.44) did not decrease the incidence of GDM. For this outcome, a large dose of probiotic bacteria was used without exception.

### 3.6. Sensitivity Analysis and Publication Risk Bias

**Figure 3:**
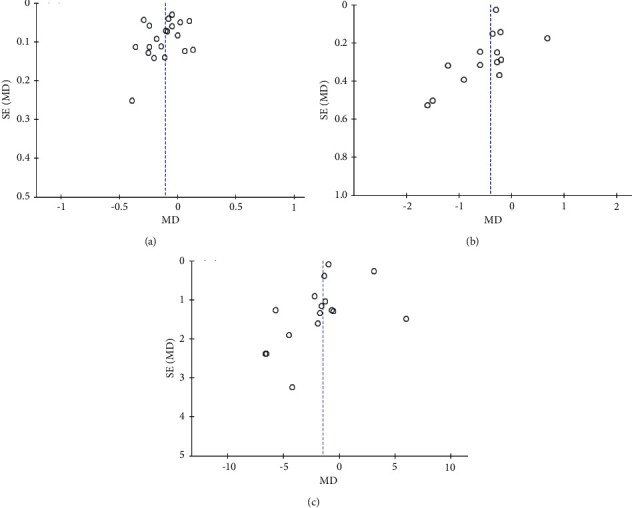
Funnel plot. (a) FPG. (b) HOMA-IR. (c) Insulin.

After the subgroup analysis, a sensitivity analysis was performed to remove several articles (Callaway et al. [33]; Pellonperä et al. [36]; Jafarnejad et al. [37]; Dolatkhah et al. [43]) that mainly contributed to substantial heterogeneity. This review largely produced similar results but better precision with less heterogeneity, and most pooled results showed relative stability (Table 3). Except for the sensitivity analyses of the FPG in the overweight/obese women subgroup and long-term intervention subgroup, the FPG and HOMA-IR in long-term intervention subgroup, all of them, had opposite results. The funnel plots of the FPG level, insulin, and HOMA-IR were also visually symmetric, which showed no significant publication risk bias. Most of the included studies focused on the top of the funnel, which further demonstrated that the sample size was large and this review is credible (Figure 3).

## 4. Discussion

The pooled analyses indicated that probiotic supplements had positive effects on improving FPG level, insulin level, insulin resistance, and insulin sensitivity, especially in GDM and healthy pregnant women. After subgroup analysis, the effects of the probiotic capsule were better than those of probiotic yogurt, short-term intervention (≤12 weeks) seemed to be more effective in glucose metabolism, and a large dose of probiotics (≥10^9^ CFU) played a role in decreasing FPG. However, probiotic supplements neither improved the level of HbA1c and 1 h and 2 h OGTT nor reduced the incidence of GDM.

Compared with previous systematic reviews, this present review included more RCTs and conducted several subgroups to control clinical heterogeneity, including different pregnancy status, duration of probiotic intervention, intervention types, and dosages, except for when grouping studies by probiotic species, because each combination of probiotic chains was only utilized in one or two studies. Sensitivity analyses were also performed to confirm the improved credibility of the results. Furthermore, this review carefully evaluated the characteristics and qualities of each included study and concluded cautiously.

Probiotic supplements had a certain function in improving the level of FPG in this review, though it was discrepant in different pregnant women. Based on this outcome, there was no meta-analysis discussing the effects of probiotics on the improvement of blood glucose in obese pregnant women. The meta-analysis results of this study illustrated that probiotics seem to only affect blood glucose in women with GDM or healthy women. This was consistent with Barengolts et al. [52], who pointed out no evidence to prove that probiotics could help control glucose in obese participants. The probable reason for this finding could be the varied composition of the gut microbiome according to the states of gestation [53, 54]. Mokkala et al. pointed out that the inflexibility of gut microbiota may influence the probiotics to regulate glucose metabolism [55]. The gut microbiota in early pregnancy is comparable to that in nonpregnancy women. Women with GDM have lower biodiversity of the intestinal microbiota in the first trimester. The diversity of gut microbiota among prepregnancy obese pregnant women is the lowest in the early and third trimesters of pregnancy [53]. However, the result of the overweight/obese women subgroup changed obviously after sensitivity analysis. This might be contributed by one dubious study [33] that had contrary findings. Therefore, it is still unclear whether probiotics can improve blood sugar in overweight/obese pregnant women. Of particular interest is that pregnancy decreased insulin sensitivity and increased insulin, insulin resistance, and HbA1c compared with those in nonpregnancy women. Thus, the effect of probiotics could be limited among pregnant women. Additionally, the subgroup analysis of different duration of probiotic intervention found that short-term (≤12 weeks) probiotic had more positive effects on improving FPG than did long-term intervention (>12 weeks). Similarly, short-term intervention could affect FPI and HOMA-IR, which is opposite to the meta-analysis results of Han et al. [56]. With the advance of gestation, the gut microbiota associated with inflammatory states is increased substantially in healthy pregnant women. Therefore, probiotic supplements might be more helpful to control blood glucose in the short term. The available studies only performed subgroup analyses of different intervention forms in FPG. Probiotic capsules were a better choice than probiotic yogurt in the level of FPG because only a certain dose of probiotics can confer a benefit, and probiotic capsules have a more accurate dose compared with probiotic yogurt. The traditional storage method of adding probiotics to dairy products or food limits the survival of probiotics [57]. The advanced technology of making capsules ensures probiotic survival, including drying technology, microencapsulation of probiotic bacteria, improved encapsulating material, capsule size, and structure [57–59]. Intestinal flora plays an important role in energy metabolism (including diabetes and obesity) [22, 23]. There is no evidence indicating that the best dosage of probiotics can regulate blood glucose, although it is universally accepted that an adequate dose of probiotics could make a difference. The results of this meta-analysis suggested that probiotic bacteria equal to and more than 10^9^ CFU per day is significantly more effective than a lower dosage. While the results in this study did not show a remarkable effect on controlling glucose, the positive performance of probiotics cannot be denied. Thus, probiotics are expected to be an assistant treatment strategy for diabetes.

The specific mechanism of probiotics remains elusive. An increasing number of researches demonstrate that microbes and metabolites work together with metabolic function and human health. Probiotics can improve metabolism by regulating the gut microbiota that will produce numerous organic compound metabolites, such as SCFAs [49] and bile acids [60]. They are the key point in the development of metabolic diseases and the improvement of insulin sensitivity, energy metabolism, and appetite suppression [21]. The imbalance of the intestinal flora may contribute to the abnormal absorption of lipopolysaccharides and increase aberrant circulating levels of SCFAs and bile acids [21, 61]. Therefore, dysbacteriosis is a vital factor of metabolic diseases like obesity, GDM, and insulin resistance [13]. Probiotics can work through the following three mechanisms: firstly, probiotics interact with gut flora and consequently produce metabolites like SCFAs; secondly, probiotics improve the intestinal epithelial barrier; and thirdly, probiotics regulate the secretion of proinflammatory mediators like tumor necrosis factor-*α*, interleukin-6, and intestinal glucagon-like peptide 1. The reduction of them can improve glycemic control and insulin sensitivity [19, 56, 62]. Therefore, probiotic supplements are expected to be a promising approach for glucose metabolism.

Probiotic supplements can improve insulin level, *β*-cell function, insulin resistance, and insulin sensitivity, especially in healthy pregnant women or those with GDM in this study. These results resembled those of several previous systematic reviews [24–29]. In particular, we found that probiotic supplements had no positive effects on insulin and HOMA-IR among overweight/obese women. Therefore, it is necessary to conduct more research to confirm this result. Meanwhile, this review did not conduct a meta-analysis of HOMA-B and QUICKI for overweight/obese women owing to a lack of related data. In addition, the probiotic capsule, which was the most applied probiotic form in this review, had effective improvement on insulin, HOMA-IR, HOMA-B, and QUICKI. Correspondingly, both small dose and large dose had positive effects. Moreover, a short-term intervention (≤12 weeks) could make more of a difference instead of a long-term intervention.

Supplementing probiotics did not have positive effects on reducing the incidence of GDM in this study, no matter the status of the pregnant women and how much probiotics they took. In the previous conclusion, the effects of improving blood glucose were mild in healthy women and barely reduced FPG among overweight/obese pregnant women. Hence, probiotics may not be an ideal preventive strategy.

This review has several limitations. Firstly, even though both English and Chinese literature were searched, only English literature was finally included. Secondly, most of the included studies came from Iran, but some full texts from Iran could not be accessed, which might contribute to risk bias in the results and affect the generality of this study. Thirdly, there was high-level heterogeneity in the meta-analysis process which came from participants in different pregnancy status as well as the difference in duration, forms, species, and doses of the probiotic interventions. Meanwhile, given the limitations of the original article, even though participants were divided into overweight/obese women, women with GDM, and healthy pregnant women, there were still women who were concurrent with overweight/obese and GDM, which contributed to some heterogeneity for results. Moreover, the beginning and termination of intervention of the included studies differed, which might, to a certain extent, affect the results. Fourthly, outcome measurements only included glucose metabolism, insulin sensitivity, and the incidence of GDM; other maternal and adverse outcomes were not included in this review, which might lead to a limitation. Finally, while this current meta-analysis was not registered and may have small deviations, we still strictly followed the process of the systematic review.

In conclusion, probiotics could modulate blood sugar and improve insulin level within a certain range in healthy and GDM women instead of overweight/obese pregnant women. There were no related meta-analyses discussing the effects of probiotics among obese/overweight pregnant women. The specific mechanism that causes this difference among pregnant women in normal and pathological pregnancies remains unknown. One of the possibilities may be that the richness, diversity, and sensitivity of intestinal flora interfere with the role of probiotics, thereby calling for more high-quality trials with a larger number of participants to explore the effects of probiotics in obese pregnant women. A significant reduction was likewise observed for metabolism parameters in taking short-term probiotics (≤12 weeks). A dosage of 10^9^ CFU per day or more could be considered an effective dose to modulate FPG. It is suggested that the regulatory effects of probiotics are short-lived. Furthermore, probiotic capsules might be more effective than probiotic yogurt in terms of FPG level. However, probiotic supplements neither improve the levels of HbA1c and 1 h and 2 h OGTT nor reduced the incidence of GDM. The optimal probiotic dose in this study is also still unclear. Further high-quality, multicentre, and large-scale RCTs are needed to ensure the safety of probiotics, probe into the positive effects on pregnant women and infants, and confirm the dosage and duration of probiotic supplements. Additional research can be conducted to further determine the effects of probiotics on insulin resistance and insulin sensitivity among overweight/obese women.

## Figures and Tables

**Table 1:** Characteristics of included studies.

Article, year (country)	Participants characteristics	Mean age, BMI (intervention/control)	Intervention/control (sample size)	Probiotic species and dose	Frequency and duration	Outcomes
Okesene-Gafa et al., 2019 (New Zealand) [32]	Pregnant women with obesity	28.9 ± 5.7/28.6 ± 5.7, 38.9 ± 6.5/38.2 ± 5.7	Probiotic/placebo (115/115)	*Lactobacillus rhamnosus* LGG and *Bifidobacterium lactis* Bb12 (6.5 × 10^9^ CFU)	1 capsule daily from 12^+0^–17^+6^ weeks of gestation until delivery	OGTT, HbA1c, and incident of GDM
Callaway et al., 2019 (Australia) [33]	Overweight or obese pregnant women	31.3 ± 4.7/31.7 ± 4.8, 31.9 ± 7.5/31.6 ± 7.2	Probiotic/placebo (219/214)	*Lactobacillus rhamnosus* LGG and *Bifidobacterium animalis* subspecies *lactis* Bb12 (>1 × 10^9^ CFU)	1 capsule daily from enrolment (15.9 ± 1.9) until delivery	OGTT and incident of GDM
Lindsay et al., 2014 (Ireland) [34]	Pregnant women with obesity	31.4 ± 5.0/31.0 ± 5.2, 32.9 ± 2.4/34.1 ± 2.7	Probiotic/placebo (64/76)	*Lactobacillus salivarius* UCC118 (10^9^ CFU)	1 capsule daily from 24 weeks to 28 weeks of gestation	FBG, insulin, HOMA-IR, and incident of GDM
Asgharian et al., 2020 (Iran) [35]	Pregnant women with obesity	29.5 ± 6.2/29.4 ± 5.5, 29.2 ± 3.3/30.3 ± 4.1	Probiotic yogurt/conventional yogurt (83/82)	*Lactobacillus acidophilus* La5 and *Bifidobacterium lactis* Bb12 (10^9^ CFU)	100 g/day from 24 weeks of gestation until delivery	FPG, OGTT, and incident of GDM
Pellonperä et al., 2019 (Finland) [36]	Overweight or obese pregnant women	30.4 ± 4.8/30.8 ± 4.8/30.8 ± 4.6/30.4 ± 4.1, 30.0 ± 4.2/29.9 ± 4.7/29.3 ± 3.9/29.7 ± 4.2	Fish oil + placebo/probiotics + placebo/fish oil + probiotics/placebo + placebo(110/109/109/110)	*Lactobacillus rhamnosus* HN001 and *Bifidobacterium animalis* ssp. *lactis* 420 (10^10^ CFU)	Daily consumption from randomization (12.5 ± 3.1) until delivery	OGTT, FPG, insulin, HOMA-IR, and incident of GDM
Jafarnejad et al., 2016 (Iran) [37]	Pregnant women with GDM	32.4 ± 3.1/31.9 ± 4.0, 26.8 ± 2.7/27.4 ± 3.1	Probiotic/placebo (41/41)	*Streptococcus thermophilus*, *Bifidobacterium breve*, *Bifidobacterium longum*, *Bifidobacterium infantis*, *Lactobacillus acidophilus*, *Lactobacillus plantarum*, *Lactobacillus paracasei*, and *Lactobacillus delbrueckii* (112.5 × 10^9^ CFU)	Twice per day from 16 weeks of gestation for 8 weeks	FPG, HbA1c, insulin, and HOMA-IR
Karamali, 2016 (Iran) [38]	Pregnant women with GDM	31.8 ± 6.0/29.7 ± 4.0, 28.6 ± 4.2/28.5 ± 3.4	Probiotic/placebo(30/30)	*Lactobacillus acidophilus*, *Lactobacillus casei*, and *Bifidobacterium bifidum* (6 × 10^9^ CFU/g)	A daily capsule from diagnosis for 6 weeks	FPG, insulin, HOMA-IR, HOMA-B, and QUICKI
Lindsay et al., 2015 (Ireland) [39]	Pregnant women with GDM	33.5 ± 5.0/32.6 ± 4.5, 29.06 ± 6.70/28.94 ± 5.79	Probiotic/placebo (74/75)	*Lactobacillus salivarius* UCC118 (10^9^ CFU)	1 capsule daily from diagnosis until delivery	FPG, insulin, and HOMA-IR
Ahmadi et al., 2016 (Iran) [40]	Pregnant women with GDM	28.5 ± 5.8/28.7 ± 3.4, 28.7 ± 4.5/28.4 ± 2.7	Symbiotic/placebo (35/35)	*Lactobacillus acidophilus*, *Lactobacillus casei*, and *Bifidobacterium bifidum* (6 × 10^9^ CFU/g)	A daily capsule from diagnosis for 6 weeks	FPG, insulin, HOMA-IR, HOMA-B, and QUICKI
Nabhani et al., 2018 (Iran) [41]	Pregnant women with GDM	29.4 ± 5.8/30.3 ± 5.6, 26.4 ± 4.1/28.2 ± 4.7	Symbiotic/placebo (47/48)	*L. acidophilus* (5 × 10^10^ CFU/g), *L. plantarum* (1.5 × 10^10^ CFU/g), *L. fermentum* (7 × 10^9^ CFU/g), and *L. gasseri* (2 × 10^10^ CFU/g)	A daily capsule from diagnosis for 6 weeks	FPG, insulin, HOMA-IR, and QUICKI
Kijmanawat et al., 2019 (Thailand) [42]	Pregnant women with GDM	32.50 ± 5.02/30.72 ± 5.05,-	Probiotic/placebo (30/30)	*Lactobacillus acidophilus* and *Bifidobacterium bifidum (*2 × 10^10^ CFU)	1 capsule daily for 4 weeks	FPG, insulin, and HOMA-IR
Dolatkhah et al., 2015 (Iran) [43]	Pregnant women with GDM	28.14 ± 6.24/26.48 ± 5.23, 31.41 ± 3.92/29.86 ± 3.39	Probiotic/placebo (32/32)	*Lactobacillus acidophilus* (La5), *Bifidobacterium* (Bb12), *Streptococcus thermophilus* (STY-31), and *Lactobacillus delbrueckii bulgaricus* (LBY-27) (>4 × 10^9^ CFU)	1 capsule daily from diagnosis for 8 weeks	FPG, insulin, HOMA-IR, and QUICKI
Babadi et al., 2019 (Iran) [44]	Pregnant women with GDM	28.8 ± 4.3/29.0 ± 4.226.1 ± 2.2/26.5 ± 2.7	Probiotic/placebo (25/25)	*Lactobacillus acidophilus, Lactobacillus casei, Bifidobacterium bifidum,* and *Lactobacillus fermentum* (8 × 10^9^ CFU/g)	1 capsule daily for 6 weeks	FPG insulin, HOMA-IR, and QUICKI
Jamilian et al., 2018 (Iran) [45]	Pregnant women with GDM	31.2 ± 5.9/29.9 ± 3.7 26.4 ± 4.2/27.5 ± 3.3	Probiotic/placebo (30/30)	*Lactobacillus acidophilus, Bifidobacterium bifidum, L. reuteri,* and *Lactobacillus fermentum* (8 × 10^9^ CFU/g)	Daily capsule for 6 weeks	FPG, insulin, HOMA-IR, and QUICKI
Sahhaf et al., 2019 (Iran) [46]	Pregnant women with GDM	31.64 ± 5.97/31.61 ± 5.49, 31.67 ± 5.44/29.67 ± 3.03	Probiotic yogurt/conventional yogurt (42/42)	*Lactobacillus acidophilus* and *Bifidobacterium lactis* (10^6^ CFU)	300 mg/day for 8 weeks	FPG and HbA1c
Taghizadeh et al., 2014 (Iran) [47]	Healthy pregnant women	26.4 ± 6.3/29.0 ± 4.6, 27.9 ± 5.1/28.2 ± 4.1	Symbiotic food/control food (28/28)	*Lactobacillus sporogenes* (1 × 10^7^ CFU/g)	Twice times per day with 18 g for 9 weeks	FPG, insulin, HOMA-IR, HOMA-B, and QUICKI
Jamilian et al., 2016 (Iran) [48]	Healthy pregnant women	27.1 ± 5.1/28.4 ± 5.3, 25.6 ± 4.2/25.5 ± 4.1	Probiotic/placebo (30/30)	*Lactobacillus acidophilus, Lactobacillus casei,* and *Bifidobacterium bifidum* (6 × 10^9^ CFU/g)	1 capsule daily from 9 weeks of gestation for 12 weeks	FPG, insulin, HOMA-IR, HOMA-B, and QUICKI
Wickens et al., 2017 (New Zealand) [49]	Pregnant women with a personal or partner history of atopic disease	34 ± 4/34 ± 4, 25 ± 4/26 ± 5	Probiotic/placebo (212/211)	*Lactobacillus rhamnosus* HN001 (6 × 10^9^ CFU)	A daily capsule from 14 to 16 weeks of gestation throughout pregnancy and until 6 months after birth if still breast-feeding	OGTT and incident of GDM
Asemi et al., 2013 (Iran) [50]	Healthy pregnant women	18-30, -	Probiotic yogurt/conventional yogurt (42/40)	*Lactobacillus acidophilus* La5 and *Bifidobacterium animalis* Bb12 (1 × 10^7^ CFU)	200 g/day for 9 weeks	FPG, insulin, and HOMA-IR
Laitinen et al., 2009 (Finland) [51]	Healthy pregnant women	29.7 ± 4.1/30.1 ± 5.2, -	Diet + probiotic/diet + placebo (85/86)	*Lactobacillus rhamnosus* LGG and *Bifidobacterium lactis* Bb12 (2 × 10^10^ CFU)	A daily capsule from the first visit (13.9 ± 1.6) until the end of exclusive breast-feeding	Insulin, HOMA-IR, and QUICKI

GDM, gestational diabetes mellitus; GWG, gestational weight gain; CFU, colony-forming unit; HOMA-IR, homeostasis model assessment insulin resistance; HOMA-B, homeostasis model of assessment-estimated *β* cell function; FBG, fasting blood glucose; FPG, fasting plasma glucose; HbA1c, glycated Hb; BMI, body mass index; QUICKI, the quantitative insulin sensitivity check index; IGT, impaired glucose tolerance; OGTT, 75 g oral glucose tolerance test.

**Table 2:** Meta-analysis of the effects of probiotics in different subgroups.

Outcome or subgroup	Studies	N	Statistical method	Effect estimate	*P*	*I*^2^ (%)
*FPG*						
FPG	20	2555	Mean difference (IV, random, 95% CI)	−0.11 [−0.17, −0.04]	0.0007	72
*Different pregnancy status for FPG*						
Overweight/obesity	6	1233	Mean difference (IV, random, 95% CI)	−0.09 [−0.20, 0.02]	0.12	78
GDM	10	740	Mean difference (IV, random, 95% CI)	−0.12 [−0.24, −0.01]	0.04	76
Healthy	4	579	Mean difference (IV, random, 95% CI)	−0.07 [−0.11, −0.02]	0.004	0
*Different intervention durations for FPG*						
Short term	12	827	Mean difference (IV, random, 95% CI)	−0.13 [−0.22, −0.04]	0.006	74
Long term	8	1725	Mean difference (IV, random, 95% CI)	−0.08 [−0.17, 0.00]	0.06	72
*Different intervention forms for FPG*						
Yogurt	3	282	Mean difference (IV, random, 95% CI)	−0.09 [−0.18, 0.00]	0.05	26
Food	1	52	NA	NA	NA	NA
Capsule	16	2221	Mean difference (IV, random, 95% CI)	−0.10 [−0.17, −0.03]	0.008	77
*Different probiotic bacteria doses for FPG*						
Small dose	3	206	Mean difference (IV, random, 95% CI)	−0.11 [−0.25, 0.04]	0.15	29
Large dose	17	2349	Mean difference (IV, random, 95% CI)	−0.10 [−0.17, −0.03]	0.004	75
*1 h OGTT*						
1 h OGTT	6	1434	Mean difference (IV, fixed, 95% CI)	−0.07 [−0.25, 0.10]	0.42	0
*Different pregnancy status for 1 h OGTT*						
Overweight/obesity	5	1060	Mean difference (IV, fixed, 95% CI)	−0.04 [−0.24, 0.16]	0.71	11
Healthy	1	374	NA	NA	NA	NA
*Different intervention forms for 1 h OGTT*						
Yogurt	1	125	NA	NA	NA	NA
Capsule	5	1309	Mean difference (IV, fixed, 95% CI)	0.23 [−0.24, 0.70]	0.75	0
*2 h OGTT*						
2 h OGTT	6	1491	Mean Difference (IV, Fixed, 95% CI)	−0.03 [−0.17, 0.12]	0.72	45
*Different pregnancy status for 2 h OGTT*						
Overweight/obesity	5	1097	Mean difference (IV, fixed, 95% CI)	0.01 [−0.15, 0.18]	0.89	52
Healthy	1	394	NA	NA	NA	NA
*Different intervention forms for 2 h OGTT*						
Yogurt	1	118	NA	NA	NA	NA
Capsule	5	1373	Mean difference (IV, fixed, 95% CI)	0.03 [−0.11, 0.18]	0.65	0
*HbA1c*						
HbA1c	3	389	Mean difference (IV, fixed, 95% CI)	−0.05 [−0.12, 0.03]	0.23	0
*Insulin level*						
Insulin	16	1446	Mean difference (IV, random, 95% CI)	−1.68 [−2.44, −0.92]	<0.00001	76
*Different pregnancy status for insulin*						
Overweight/obesity	3	502	Mean difference (IV, random, 95% CI)	0.94 [−3.36, 5.24]	0.67	90
GDM	9	605	Mean difference (IV, random, 95% CI)	−2.40 [−3.70, −1.09]	0.0003	70
Healthy	4	339	Mean difference (IV, random, 95% CI)	−1.77 [−2.40, −1.14]	<0.00001	24
*Different intervention durations for insulin*						
Short term	12	825	Mean difference (IV, random, 95% CI)	−2.27 [−3.13, −1.41]	<0.00001	72
Long term	4	621	Mean difference (IV, random, 95% CI)	0.42 [−2.34, 3.18]	0.77	87
*Different intervention forms for insulin*						
Yogurt	1	70	NA	NA	NA	NA
Food	1	52	NA	NA	NA	NA
Capsule	14	1324	Mean difference (IV, random, 95% CI)	−1.66 [−2.59, −0.73]	0.0005	76
*Different probiotic bacteria doses for insulin*						
Small dose	2	122	Mean difference (IV, fixed, 95% CI)	−1.92 [−2.48, −1.36]	<0.00001	0
Large dose	14	1423	Mean difference (IV, fixed, 95% CI)	−1.04 [−1.22, −0.85]	<0.00001	76
*HOMA-IR level*						
HOMA-IR	16	1383	Mean difference (IV, random, 95% CI)	−0.36 [−0.53, −0.20]	<0.00001	74
*Different pregnancy status for HOMA-IR*						
Overweight/obesity	3	502	Mean difference (IV, random, 95% CI)	0.07 [−0.55, 0.70]	0.81	89
GDM	9	610	Mean difference (IV, random, 95% CI)	−0.59 [−0.88, −0.30]	<0.00001	64
Healthy	4	271	Mean difference (IV, random, 95% CI)	−0.34 [−0.48, −0.20]	<0.00001	7
*Different intervention durations for HOMA-IR*						
Short term	12	830	Mean difference (IV, random, 95% CI)	−0.47 [−0.64, −0.31]	<0.00001	57
Long term	4	553	Mean difference (IV, random, 95% CI)	−0.02 [−0.51, 0.47]	0.94	87
*Different intervention forms for HOMA-IR*						
Yogurt	1	70	NA	NA	NA	NA
Food	1	52	NA	NA	NA	NA
Capsule	14	1261	Mean difference (IV, random, 95% CI)	−0.40 [−0.63, −0.18]	0.0004	77
*Different probiotic bacteria doses for HOMA-IR*						
Small dose	2	251	Mean difference (IV, random, 95% CI)	−0.29 [−0.41, −0.17]	<0.00001	94
Large dose	14	1261	Mean difference (IV, random, 95% CI)	−0.29 [−0.34, −0.24]	<0.00001	77
*HOMA-B*						
HOMA-B	4	242	Mean difference (IV, fixed, 95% CI)	−21.80 [−31.92, −11.67]	<0.00001	0
*Different pregnancy status for HOMA-B*						
GDM	2	130	Mean difference (IV, fixed, 95% CI)	−25.25 [−39.04, −11.47]	0.0003	0
Healthy	2	112	Mean difference (IV, fixed, 95% CI)	−17.74 [−32.66, −2.82]	0.02	0
*Different intervention forms for HOMA-B*						
Food	1	52	NA	NA	NA	NA
Capsule	3	190	Mean difference (IV, fixed, 95% CI)	−22.76 [−33.32, −12.19]	<0.00001	0
*Different probiotic bacteria doses for HOMA-B*						
Small dose	1	52	NA	NA	NA	NA
Large dose	3	190	Mean difference (IV, fixed, 95% CI)	−22.76 [−33.32, −12.19]	<0.00001	0
*QUICKI*						
QUICKI	9	582	Mean difference (IV, fixed, 95% CI)	0.01 [0.00, 0.01]	0.001	48
*Different pregnancy status for QUICKI*						
GDM	6	381	Mean difference (IV, fixed, 95% CI)	0.00 [0.00, 0.01]	0.03	49
Healthy	3	201	Mean difference (IV, fixed, 95% CI)	0.02 [0.01, 0.03]	0.001	0
*Different intervention durations for QUICKI*						
Short term	8	493	Mean difference (IV, fixed, 95% CI)	0.00 [0.00, 0.01]	0.02	28
Long term	1	89	NA	NA	NA	NA
*Different intervention forms for QUICKI*						
Food	1	52	NA	NA	NA	NA
Capsule	8	530	Mean difference (IV, fixed, 95% CI)	0.01 [0.00, 0.01]	0.002	55
*Different probiotic bacteria doses for QUICKI*						
Small dose	1	52	NA	NA	NA	NA
Large dose	8	530	Mean difference (IV, fixed, 95% CI)	0.01 [0.00, 0.01]	0.002	55
*Incidence of GDM*						
The incident rate of GDM	7	1645	Risk ratio (M-H, fixed, 95% CI)	1.03 [0.94, 1.18]	0.67	37
*Different pregnancy status for the incident of GDM*						
Overweight/obesity	6	1272	Risk ratio (M-H, fixed, 95% CI)	1.09 [0.95, 1.25]	0.24	4
Healthy	1	373	NA	NA	NA	NA
*Different intervention durations for the incident of GDM*						
Short term	1	136	NA	NA	NA	NA
Long term	6	1508	Risk ratio (M-H, fixed, 95% CI)	1.03 [0.90, 1.18]	0.69	48
*Different intervention forms for the incident of GDM*						
Yogurt	1	128	NA	NA	NA	NA
Capsule	6	1516	Risk ratio (M-H, fixed, 95% CI)	1.05 [0.92, 1.21]	0.44	31

NA, not available.

**Table 3:** Sensitivity analysis on effects of probiotic supplement on glucose metabolism.

Outcome	Before sensitivity analysis			Remove study	After sensitivity analysis		
	Effect estimate	*P*	*I*^2^ (%)		Effect estimate	*P*	*I*^2^ (%)
*FPG*							
FPG level	−0.11 [−0.17, −0.04]	0.0007	72	Callaway et al., 2019; Dolatkhah et al., 2015	−0.10 [−0.15, −0.05]	<0.00001	46
*Different pregnancy status for FPG*							
Overweight/obesity	−0.09 [−0.20, 0.02]	0.12	78	Callaway et al., 2019	−0.13 [−0.21, −0.06]	0.0006	34
GDM	−0.12 [−0.24, −0.01]	0.04	76	Jafarnejad et al., 2016; Lindsay et al., 2015; Nabhani et al., 2018	−0.22 [−0.32, −0.13]	<0.00001	35
*Different intervention durations for FPG*							
Short term	−0.13 [−0.22, −0.04]	0.006	74	Dolatkhah et al., 2015; Jafarnejad et al., 2016	−0.12 [−0.20, −0.03]	0.007	48
Long term	−0.08 [−0.17, 0.00]	0.06	72	Callaway et al., 2019	−0.11 [−0.18, −0.05]	0.0005	35
*Different intervention forms for FPG*							
Capsule	−0.10 [−0.17, −0.03]	0.008	77	Callaway et al., 2019; Dolatkhah et al., 2015; Pellonperä et al., 2019B	−0.08 [−0.13, −0.02]	0.008	38
*Different probiotic bacteria doses for FPG*							
Large dose	−0.10 [−0.17, −0.03]	0.004	75	Callaway et al., 2019; Dolatkhah et al., 2015; Jafarnejad et al., 2016	−0.12 [−0.17, −0.06]	<0.0001	41
*2 h OGTT*							
*Different pregnancy status for 2 h OGTT*							
Overweight/obesity	0.01 [−0.15, 0.18]	0.89	52	Asgharian et al., 2020	0.10 [−0.07, 0.27]	0.26	0
*Insulin*							
Insulin	−1.68 [−2.44, −0.92]	<0.00001	76	Jafarnejad et al., 2016; Karamali et al., 2016; Pellonperä et al., 2019B	−1.52 [−2.03, −1.02]	<0.00001	44
*Different pregnancy status for insulin*							
Overweight/obesity	0.94 [−3.36, 5.24]	0.67	90	Pellonperä et al., 2019A	−1.41 [−2.93, 0.12]	0.07	0
GDM	−2.40 [−3.70, −1.09]	0.0003	70	Ahmadi et al., 2016; Jafarnejad et al., 2016; Karamali, 2016	−0.99 [−1.18, −0.80]	<0.00001	0
*Different intervention durations for insulin*							
Short term	−2.27 [−3.13, −1.41]	<0.00001	72	Ahmadi et al., 2016; Jafarnejad et al., 2016; Karamali, 2016	−1.55 [−2.17, −0.93]	<0.00001	49
Long term	0.42 [−2.34, 3.18]	0.77	87	Pellonperä et al., 2019B	−1.32 [−2.02, −0.63]	0.0002	0
*Different types of intervention for insulin*							
Capsule	−1.66 [−2.59, −0.73]	0.0005	76	Jafarnejad et al., 2016; Pellonperä et al., 2019B	−1.52 [−2.14, −0.91]	<0.00001	38
*Different probiotic bacteria doses for insulin*							
Large dose	−1.04 [−1.22, −0.85]	<0.00001	76	Jafarnejad et al., 2016; Pellonperä et al., 2019B	−1.04 [−1.23, −0.86]	<0.00001	38
*HOMA-IR*							
HOMA-IR	−0.36 [−0.53, −0.20]	<0.00001	74	Pellonperä et al., 2019B; Taghizadeh et al., 2014	−0.39 [−0.52, −0.27]	<0.00001	49
*Different pregnancy status for HOMA-IR*							
Overweight/obesity	0.07 [−0.55, 0.70]	0.81	89	Pellonperä et al., 2019B	−0.23 [−0.47, 0.02]	0.07	0
GDM	−0.59 [−0.88, −0.30]	<0.00001	64	Dolatkhah et al., 2015; Jafarnejad et al., 2016	−0.59 [−0.93, −0.25]	0.0007	44
*Different intervention durations for HOMA-IR*							
Short term	−0.45 [−0.62, −0.29]	<0.00001	57	Karamali et al., 2016	−0.42 [−0.57, −0.28]	<0.00001	49
Long term	−0.15 [−0.61, 0.32]	0.53	85	Pellonperä et al., 2019B	−0.27 [−0.47, −0.08]	0.006	0
*Different types of intervention for HOMA-IR*							
Capsule	−0.40 [−0.63, −0.18]	0.0004	77	Jafarnejad et al., 2016; Pellonperä et al., 2019B	−0.39 [−0.54, −0.24]	<0.00001	37
*Different probiotic bacteria doses for HOMA-IR*							
Large dose	−0.29 [−0.34, −0.24]	<0.00001	77	Karamali, 2016; Pellonperä et al., 2019B	−0.30 [−0.35, −0.26]	<0.00001	43
*QUIICKI*							
*Different types of intervention for QUIICKI*							
Capsule	0.01 [0.00, 0.01]	0.002	55	Babadi et al., 2019	0.01 [0.01, 0.02]	<0.00001	0
*Different intervention durations for the incident of GDM*							
Long term	1.05 [0.81, 1.36]	0.69	52	Wickens et al., 2017	1.18 [0.89, 1.57]	0.24	36

## Data Availability

All the data of the review are available from public electronic databases.

## Abbreviations

GDM: Gestational diabetes mellitus
RCT: Randomized controlled trial
OGTT: 75 g oral glucose tolerance test
CI: Confidence interval
MD: Mean difference
HOMA-IR: Homeostasis model assessment insulin resistance
HOMA-B: Homeostasis model of assessment-estimated *β* cell function
FBG: Fasting blood glucose
FPG: Fasting plasma glucose
HbA1c: Glycated Hb
SCFAs: Short-chain fatty acids
BMI: Body mass index
QUICKI: The quantitative insulin sensitivity check index
GWG: Gestational weight gain
CFU: Colony-forming unit
IGT: Impaired glucose tolerance.

## Supplementary Materials

Supplementary Materials Supplementary file 1: search strategy for each database used in this review. Supplementary file 2: PRISMA 2009 checklist.

## References

[1] Pérez-Pérez A., Vilariño-García T., Guadix P., Dueñas J. L., Sánchez-Margalet V. (2020). Leptin and nutrition in gestational diabetes. *Nutrients*.

[2] McIntyre H. D., Catalano P., Zhang C., Desoye G., Mathiesen E. R., Damm P. (2019). Gestational diabetes mellitus. *Nature Reviews Disease Primers*.

[3] Gao C., Sun X., Lu L., Liu F., Yuan J. (2019). Prevalence of gestational diabetes mellitus in mainland China: a systematic review and meta-analysis. *Journal of Diabetes Investigation*.

[4] Kim S. Y., England L., Wilson H. G., Bish C., Satten G. A., Dietz P. (2010). Percentage of gestational diabetes mellitus attributable to overweight and obesity. *American Journal of Public Health*.

[5] Neeland I. J., Poirier P., Després J.-P. (2018). Cardiovascular and metabolic heterogeneity of obesity. *Circulation*.

[6] Powe C. E., Allard C., Battista M.-C. (2016). Heterogeneous contribution of insulin sensitivity and secretion defects to gestational diabetes mellitus: table 1. *Diabetes Care*.

[7] Catalano P. M., McIntyre H. D., Cruickshank J. K. (2012). The hyperglycemia and adverse pregnancy outcome study: associations of GDM and obesity with pregnancy outcomes. *Diabetes Care*.

[8] LifeCycle Project-Maternal Obesity and Childhood Outcomes Study Group, Voerman E., Voerman E. (2019). Association of gestational weight gain with adverse maternal and infant outcomes. *Journal of the American Medical Association*.

[9] Dickens L. T., Thomas C. C. (2019). Updates in gestational diabetes prevalence, treatment, and health policy. *Current Diabetes Reports*.

[10] Dall T. M., Yang W., Gillespie K. (2019). The economic burden of elevated blood glucose levels in 2017: diagnosed and undiagnosed diabetes, gestational diabetes mellitus, and prediabetes. *Diabetes Care*.

[11] Cremona A., Saunders J., Cotter A., Hamilton J., Donnelly A. E., O’Gorman C. S. (2020). Maternal obesity and degree of glucose intolerance on neonatal hypoglycaemia and birth weight: a retrospective observational cohort study in women with gestational diabetes mellitus. *European Journal of Pediatrics*.

[12] Lowe W. L., Scholtens D. M., Kuang A. (2019). Hyperglycemia and adverse pregnancy outcome follow-up study (HAPO FUS): maternal gestational diabetes mellitus and childhood glucose metabolism. *Diabetes Care*.

[13] Buchanan T. A., Xiang A. H., Page K. A. (2012). Gestational diabetes mellitus: risks and management during and after pregnancy. *Nature Reviews Endocrinology*.

[14] Spaight C., Gross J., Horsch A., Puder J. J. (2016). Gestational diabetes mellitus. *Endocrine Development*.

[15] Martis R., Brown J., McAra-Couper J., Crowther C. A. (2018). Enablers and barriers for women with gestational diabetes mellitus to achieve optimal glycaemic control - a qualitative study using the theoretical domains framework. *BMC Pregnancy and Childbirth*.

[16] Zulfiqar T., Lithander F. E., Banwell C. (2017). Barriers to a healthy lifestyle post gestational-diabetes: an Australian qualitative study. *Women and Birth*.

[17] Ge L., Albin B., Hadziabdic E., Hjelm K., Rask M. (2016). Beliefs about health and illness and health-related behavior among urban women with gestational diabetes mellitus in the south east of China. *Journal of Transcultural Nursing*.

[18] Hill C., Guarner F., Reid G. (2014). The International Scientific Association for Probiotics and Prebiotics consensus statement on the scope and appropriate use of the term probiotic. *Nature Reviews Gastroenterology & Hepatology*.

[19] Falcinelli S., Rodiles A., Hatef A., Picchietti S., Cossignani L., Merrifield D. L. (2018). Influence of probiotics administration on gut microbiota core: a review on the effects on appetite control, glucose, and lipid metabolism. *Journal of Clinical Gastroenterology*.

[20] Sánchez-Tapia M., Tovar A. R., Torres N. (2019). Diet as regulator of gut microbiota and its role in health and disease. *Archives of Medical Research*.

[21] Meijnikman A. S., Gerdes V. E., Nieuwdorp M., Herrema H. (2018). Evaluating causality of gut microbiota in obesity and diabetes in humans. *Endocrine Reviews*.

[22] Patterson E., Ryan P. M., Cryan J. F. (2016). Gut microbiota, obesity and diabetes. *Postgraduate Medical Journal*.

[23] Stenman L. K., Burcelin R., Lahtinen S. (2016). Establishing a causal link between gut microbes, body weight gain and glucose metabolism in humans—towards treatment with probiotics. *Beneficial Microbes*.

[24] Zhang J., Ma S., Wu S., Guo C., Long S., Tan H. (2019). Effects of probiotic supplement in pregnant women with gestational diabetes mellitus: a systematic review and meta-analysis of randomized controlled trials. *Journal of diabetes research*.

[25] Taylor B., Woodfall G., Sheedy K. (2017). Effect of probiotics on metabolic outcomes in pregnant women with gestational diabetes: a systematic review and meta-analysis of randomized controlled trials. *Nutrients*.

[26] Pan J., Pan Q., Chen Y., Zhang H., Zheng X. (2019). Efficacy of probiotic supplement for gestational diabetes mellitus: a systematic review and meta-analysis. *Journal of Maternal-Fetal and Neonatal Medicine*.

[27] Zheng J., Feng Q., Zheng S., Xiao X. (2018). The effects of probiotics supplementation on metabolic health in pregnant women: an evidence based meta-analysis. *PLoS One*.

[28] Masulli M., Vitacolonna E., Fraticelli F., Della Pepa G., Mannucci E., Monami M. (2020). Effects of probiotic supplementation during pregnancy on metabolic outcomes: a systematic review and meta-analysis of randomized controlled trials. *Diabetes Research and Clinical Practice*.

[29] Peng T.-R., Wu T.-W., Chao Y.-C. (2018). Effect of probiotics on the glucose levels of pregnant women: a meta-analysis of randomized controlled trials. *Medicina*.

[30] Moher D., Liberati A., Tetzlaff J., Altman D. G., PRISMA Group (2009). Preferred reporting items for systematic reviews and meta-analyses: the PRISMA statement. *PLoS Medicine*.

[31] Cumpston M., Li T., Page M. J. (2019). Updated guidance for trusted systematic reviews: a new edition of the Cochrane Handbook for Systematic Reviews of Interventions. *Cochrane Database of Systematic Reviews*.

[32] Okesene-Gafa K. A. M., Li M., McKinlay C. J. D., Taylor R. S., Rush E. C., Wall C. R. (2019). Effect of antenatal dietary interventions in maternal obesity on pregnancy weight-gain and birthweight: healthy Mums and Babies (HUMBA) randomized trial. *American Journal of Obstetrics and Gynecology*.

[33] Callaway L. K., McIntyre H. D., Barrett H. L. (2019). Probiotics for the prevention of gestational diabetes mellitus in overweight and obese women: findings from the SPRING double-blind randomized controlled trial. *Diabetes Care*.

[34] Lindsay K. L., Kennelly M., Culliton M. (2014). Probiotics in obese pregnancy do not reduce maternal fasting glucose: a double-blind, placebo-controlled, randomized trial (Probiotics in Pregnancy Study). *American Journal of Clinical Nutrition*.

[35] Asgharian H., Homayouni-Rad A., Mirghafourvand M., Mohammad-Alizadeh-Charandabi S. (2020). Effect of probiotic yoghurt on plasma glucose in overweight and obese pregnant women: a randomized controlled clinical trial. *European Journal of Nutrition*.

[36] Pellonperä O., Mokkala K., Houttu N. (2019). Efficacy of fish oil and/or probiotic intervention on the incidence of gestational diabetes mellitus in an at-risk group of overweight and obese women: a randomized, placebo-controlled, double-blind clinical trial. *Diabetes Care*.

[37] Jafarnejad S., Saremi S., Jafarnejad F., Arab A. (2016). Effects of a multispecies probiotic mixture on glycemic control and inflammatory status in women with gestational diabetes: a randomized controlled clinical trial. *Journal of nutrition and metabolism*.

[38] Karamali M., Dadkhah F., Sadrkhanlou M. (2016). Effects of probiotic supplementation on glycaemic control and lipid profiles in gestational diabetes: a randomized, double-blind, placebo-controlled trial. *Diabetes & Metabolism*.

[39] Lindsay K. L., Brennan L., Kennelly M. A. (2015). Impact of probiotics in women with gestational diabetes mellitus on metabolic health: a randomized controlled trial. *American Journal of Obstetrics and Gynecology*.

[40] Ahmadi S., Jamilian M., Tajabadi-Ebrahimi M., Jafari P., Asemi Z. (2016). The effects of synbiotic supplementation on markers of insulin metabolism and lipid profiles in gestational diabetes: a randomised, double-blind, placebo-controlled trial. *British Journal of Nutrition*.

[41] Nabhani Z., Hezaveh S. J. G., Razmpoosh E., Asghari-Jafarabadi M., Gargari B. P. (2018). The effects of synbiotic supplementation on insulin resistance/sensitivity, lipid profile and total antioxidant capacity in women with gestational diabetes mellitus: a randomized double blind placebo controlled clinical trial. *Diabetes Research and Clinical Practice*.

[42] Kijmanawat A., Panburana P., Reutrakul S., Tangshewinsirikul C. (2019). Effects of probiotic supplements on insulin resistance in gestational diabetes mellitus: a double-blind randomized controlled trial. *Journal of Diabetes Investigation*.

[43] Dolatkhah N., Hajifaraji M., Abbasalizadeh F., Aghamohammadzadeh N., Mehrabi Y., Mesgari Abbasi M. (2015). Is there a value for probiotic supplements in gestational diabetes mellitus? A randomized clinical trial. *Journal of Health, Population and Nutrition*.

[44] Babadi M., Khorshidi A., Aghadavood E. (2019). The effects of probiotic supplementation on genetic and metabolic profiles in patients with gestational diabetes mellitus: a randomized, double-blind, placebo-controlled trial. *Probiotics and Antimicrobial Proteins*.

[45] Jamilian M., Amirani E., Asemi Z. (2019). The effects of vitamin D and probiotic co-supplementation on glucose homeostasis, inflammation, oxidative stress and pregnancy outcomes in gestational diabetes: a randomized, double-blind, placebo-controlled trial. *Clinical Nutrition*.

[46] Sahhaf Ebrahimi F., Homayouni Rad A., Mosen M., Abbasalizadeh F., Tabrizi A., Khalili L. (2019). Effect of L. acidophilus and B. lactis on blood glucose in women with gestational diabetes mellitus: a randomized placebo-controlled trial. *Diabetology & Metabolic Syndrome*.

[47] Taghizadeh M., Asemi Z. (2014). Effects of synbiotic food consumption on glycemic status and serum hs-CRP in pregnant women: a randomized controlled clinical trial. *Hormones*.

[48] Jamilian M., Bahmani F., Vahedpoor Z. (2016). Effects of probiotic supplementation on metabolic status in pregnant women: a randomized, double-blind, placebo-controlled trial. *Archives of Iranian Medicine*.

[49] Wickens K. L., Barthow C. A., Murphy R. (2017). Early pregnancy probiotic supplementation with Lactobacillus rhamnosus HN001 may reduce the prevalence of gestational diabetes mellitus: a randomised controlled trial. *British Journal of Nutrition*.

[50] Asemi Z., Samimi M., Samimi M. (2013). Effect of daily consumption of probiotic yoghurt on insulin resistance in pregnant women: a randomized controlled trial. *European Journal of Clinical Nutrition*.

[51] Laitinen K., Poussa T., Isolauri E. (2009). Nutrition, Allergy, Mucosal Immunology and Intestinal Microbiota Group. Probiotics and dietary counselling contribute to glucose regulation during and after pregnancy: a randomised controlled trial. *British Journal of Nutrition*.

[52] Barengolts E., Smith E., Reutrakul S., Tonucci L., Anothaisintawee T. (2019). The effect of probiotic yogurt on glycemic control in type 2 diabetes or obesity: a meta-analysis of nine randomized controlled trials. *Nutrients*.

[53] Koren O., Goodrich J. K., Cullender T. C. (2012). Host remodeling of the gut microbiome and metabolic changes during pregnancy. *Cell*.

[54] Taddei C. R., Cortez R. V., Mattar R., Torloni M. R., Daher S. (2018). Microbiome in normal and pathological pregnancies: a literature overview. *American Journal of Reproductive Immunology*.

[55] Mokkala K., Paulin N., Houttu N. (2020). Metagenomics analysis of gut microbiota in response to diet intervention and gestational diabetes in overweight and obese women: a randomised, double-blind, placebo-controlled clinical trial. *Gut*.

[56] Han M.-M., Sun J.-F., Su X.-H. (2019). Probiotics improve glucose and lipid metabolism in pregnant women: a meta-analysis. *Annals of Translational Medicine*.

[57] Marcial-Coba M. S., Knøchel S., Nielsen D. S. (2019). Low-moisture food matrices as probiotic carriers. *FEMS Microbiology Letters*.

[58] Šipailienė A., Petraitytė S. (2018). Encapsulation of probiotics: proper selection of the probiotic strain and the influence of encapsulation technology and materials on the viability of encapsulated microorganisms. *Probiotics Antimicrob Proteins*.

[59] Dianawati D., Mishra V., Shah N. P. (2016). Survival of microencapsulated probiotic bacteria after processing and during storage: a review. *Critical Reviews in Food Science and Nutrition*.

[60] Meng X., Zhou H.-Y., Shen H.-H. (2019). Microbe-metabolite-host axis, two-way action in the pathogenesis and treatment of human autoimmunity. *Autoimmunity Reviews*.

[61] Ponzo V., Fedele D., Goitre I. (2019). Diet-gut microbiota interactions and gestational diabetes mellitus (GDM). *Nutrients*.

[62] Isolauri E., Rautava S., Collado M. C., Salminen S. (2015). Role of probiotics in reducing the risk of gestational diabetes. *Diabetes, Obesity and Metabolism*.

