# Supplementary material for: Probiotic Supplements Improve Blood Glucose and Insulin Resistance/Sensitivity among Healthy and GDM Pregnant Women: A Systematic Review and Meta-Analysis of Randomized Controlled Trials
Source: Evid Based Complement Alternat Med. 2021 Sep 22;2021:9830200. doi: 10.1155/2021/9830200 (PMC8481047; doi:10.1155/2021/9830200)
Supplement: Supplementary Materials — Supplementary file 1: search strategy for each database used in this review. Supplementary file 2: PRISMA 2009 checklist. [file 9830200.f1.doc]

**Search Strategy**

**Mesh terms and free words**

**（1）English:**

| Concept | | Keywords | |
| --- | --- | --- | --- |
| Participants | Mesh terms | Pregnancy | Obesity. Maternal |
|  |  | Diabetes. Gestational | Overweight |
|  | Free words | pregnan* | gestation* |
|  |  | matern* | obesity |
|  |  | obstetric* | obese |
|  |  | overweight | gestational diabetes |
|  |  | gestational diabetes mellitus | GDM |
| Intervention | Mesh terms | Probiotics |  |
|  | Free words | probiotic* | symbiotic* |
|  |  | lactobacill* | streptococc* |
|  |  | bifidobacter* | saccharomy* |
|  |  | yeast |  |
| Outcomes | Mesh terms | Blood Glucose | Metabolitic Diseases |
|  | Free words | glucose | blood glucose |
|  |  | glycemic control | *glycemica |
|  |  | HbA1c | glycosylated hemoglobin A1c |
|  |  | glycated Hemoglobin A | diabetes |
|  |  | insulin | insulin diseases |
|  |  | insulin sensitivity | metabol* |

（2）Chinese:

| Concept | | Keywords | |
| --- | --- | --- | --- |
| Participants | Mesh terms | 妊娠 | 妊娠期肥胖 |
|  |  | 妊娠期糖尿病 | 超重 |
|  | Free words | 妊娠期糖尿病 | 妊娠期高血糖 |
|  |  | 孕妇 | 怀孕妇女 |
|  |  | 肥胖孕妇 | 超重孕妇 |
|  |  | 妊娠期肥胖 | 妊娠期超重 |
|  |  | 妊娠 |  |
| Intervention | Mesh terms | 益生菌 |  |
|  | Free words | 益生菌 | 益生元 |
|  |  | 乳酸菌 | 酸奶 |
|  |  | 乳酸杆菌 | 微生物制剂 |
| Outcomes | Mesh terms | 血糖 | 代谢性疾病 |
|  | Free words | 血糖 | 血糖控制 |
|  |  | 空腹血糖 | 糖化血红蛋白 |
|  |  | 糖尿病 | 胰岛素 |
|  |  | 胰岛素抵抗 | 胰岛素疾病 |

***Cochrane Library***

#1 MeSH descriptor: [Pregnancy] explode all trees

#2 MeSH descriptor: [Diabetes. Gestational] explode all trees

#3 MeSH descriptor: [Obesity. Maternal] explode all trees

#4 MeSH descriptor: [Overweight] explode all trees

#5 pregnan* or gestation* or matern* or obstetric* or expectan* or obesity or obese or overweight or gestational diabetes or gestational diabetes mellitus or GDM:ti,ab,kw (Word variations have been searched)

#6 #1 or #2 or #3 or #4 or #5

#7 MeSH descriptor: [Probiotics] explode all trees

#8 probiotic* or symbiotic* or lactobacill* or streptococc* or bifidobacter* or saccharomy* or yeast or yogurt or bacteria* or acidophilus or ferment* or microorganism* or intervention* or pharmaceutical Intervention:ti,ab,kw (Word variations have been searched)

#9 #7 or #8

#10 MeSH descriptor: [Blood Glucose] explode all trees

#11 MeSH descriptor: [Metabolitic Diseases] explode all trees

#12 glucose or blood glucose or glycemic control or *glycemica or HbA1c or metabol* glycosylated hemoglobin A1c or glycated Hemoglobin A or diabetes or insulin or insulin diseases or insulin sensitivity:ti,ab,kw (Word variations have been searched)

#13 #10 or #11 or #12

#14 #6 and #9 and #13

#15 #14 in Trials

***Embase***

#1 ' Pregnancy '/exp

#2 ' Diabetes. Gestational '/exp

#3 ' Obesity. Maternal '/exp

#4 ' Overweight '/exp

#5 pregnan*:ab,ti or gestation*:ab,ti or matern*:ab,ti or obstetric*:ab,ti or expectan*:ab,ti or obesity:ab,ti or obese:ab,ti or overweight:ab,ti or gestational diabetes:ab,ti or gestational diabetes mellitus:ab,ti or GDM:ab,ti

#6 #1 or #2 or #3 or #4 or #5

#7 ' Probiotics '/exp

#8 probiotic*:ab,ti or symbiotic*:ab,ti or lactobacill*:ab,ti or streptococc*:ab,ti or bifidobacter*:ab,ti or saccharomy*:ab,ti or yeast:ab,ti or yogurt:ab,ti or bacteria*:ab,ti or acidophilus:ab,ti or ferment*:ab,ti or microorganism*:ab,ti or intervention*:ab,ti or pharmaceutical Intervention:ab,ti

#9 #7 or #8

#10 ' Blood Glucose '/exp

#11 ' Metabolitic Diseases '/exp

#12 glucose:ab,ti or blood glucose:ab,ti or glycemic control:ab,ti or *glycemica:ab,ti or HbA1c:ab,ti or metabol*:ab,ti or glycosylated hemoglobin A1c:ab,ti or glycated Hemoglobin A:ab,ti or diabetes:ab,ti or insulin:ab,ti or insulin disease:ab,ti s or insulin sensitivity:ab,ti

#13 #10 or #11 or #12

#14 #6 and #9 and #13

***Web of Science***

#1 TOPIC: (pregnan* or gestation* or matern* or obstetric* or expectan* or obesity or obese or overweight or gestational diabetes or gestational diabetes mellitus or GDM) Indexes=SCI-EXPANDED, SSCI, A&HCI, CPCI-S, CPCI-SSH Timespan=All years

#2 TOPIC: (probiotic* or symbiotic* or lactobacill* or streptococc* or bifidobacter* or saccharomy* or yeast or yogurt or bacteria* or acidophilus or ferment* or microorganism* or intervention* or pharmaceutical Intervention) Indexes=SCI-EXPANDED, SSCI, A&HCI, CPCI-S, CPCI-SSH Timespan=All years

#3 TOPIC: (glucose or blood glucose or glycemic control or *glycemica or HbA1c or metabol* glycosylated hemoglobin A1c or glycated Hemoglobin A or diabetes or insulin or insulin diseases or insulin sensitivity) Indexes=SCI-EXPANDED, SSCI, A&HCI, CPCI-S, CPCI-SSH Timespan=All years

#4 #1 and #2 and #3

***EBSCO-Medline/ EBSCO-CINAHL***

#1 AB ( pregnan* or gestation* or matern* or obstetric* or expectan* or obesity or obese or overweight or gestational diabetes or gestational diabetes mellitus or GDM)

#2 AB ( probiotic* or symbiotic* or lactobacill* or streptococc* or bifidobacter* or saccharomy* or yeast or yogurt or bacteria* or acidophilus or ferment* or microorganism* or intervention* or pharmaceutical Intervention)

#3 AB (glucose or blood glucose or glycemic control or *glycemica or HbA1c or metabol* glycosylated hemoglobin A1c or glycated Hemoglobin A or diabetes or insulin or insulin diseases or insulin sensitivity)

#4 #1 AND #2 AND #3

***Pubmed***

#1 "Pregnancy"[MeSH Terms] OR "Diabetes, Gestational"[MeSH Terms] OR "Obesity, Maternal"[MeSH Terms] OR "Overweight"[MeSH Terms]

#2 "pregnan*"[Title/Abstract] OR "gestation*"[Title/Abstract] OR "matern*"[Title/Abstract] OR "obstetric*"[Title/Abstract] OR "expectan*"[Title/Abstract] OR "obesity"[Title/Abstract] OR "obese"[Title/Abstract] OR "overweight"[Title/Abstract] OR "gestational diabetes"[Title/Abstract] OR "gestational diabetes mellitus"[Title/Abstract] OR "GDM"[Title/Abstract]

#3 #1 or #2

#4 "Probiotics"[MeSH Terms]

#5 "probiotic*"[Title/Abstract] OR "symbiotic*"[Title/Abstract] OR "lactobacill*"[Title/Abstract] OR "streptococc*"[Title/Abstract] OR "bifidobacter*"[Title/Abstract] OR "saccharomy*"[Title/Abstract] OR "yeast"[Title/Abstract] OR "yogurt"[Title/Abstract] OR "bacteria*"[Title/Abstract] OR "acidophilus"[Title/Abstract] OR "ferment*"[Title/Abstract] OR "microorganism*"[Title/Abstract] OR "intervention*"[Title/Abstract] OR "pharmaceutical intervention"[Title/Abstract]

#6 #4 or #5

#7 "Blood Glucose"[MeSH Terms] OR " Metabolitic Diseases "[MeSH Terms]

#8 (((((((("glucose"[Title/Abstract] OR "blood glucose"[Title/Abstract]) OR "glycemic control"[Title/Abstract]) OR "HbA1c"[Title/Abstract]) OR ("metabol*"[All Fields] AND "glycosylated hemoglobin a1c"[Title/Abstract])) OR "glycated hemoglobin a"[Title/Abstract]) OR "diabetes"[Title/Abstract]) OR "insulin"[Title/Abstract]) OR ((((((((("insulin"[MeSH Terms] OR "insulin"[All Fields]) OR "insulin s"[All Fields]) OR "insuline"[All Fields]) OR "insulinic"[All Fields]) OR "insulinization"[All Fields]) OR "insulinized"[All Fields]) OR "insulins"[MeSH Terms]) OR "insulins"[All Fields]) AND "diseases"[Title/Abstract])) OR "insulin sensitivity"[Title/Abstract]

#8 #7 OR #8

#9 #3 AND #6 AND #8

***ClinicalTrials.gov***

Available, Completed Studies | Interventional Studies | pregnan* or gestation* or matern* or obstetric* or expectan* or obesity or obese or overweight or gestational diabetes or gestational diabetes mellitus or GDM | probiotic* or symbiotic* or lactobacill* or streptococc* or bifidobacter* or saccharomy* or yeast or yogurt or bacteria* or acidophilus or ferment* or microorganism* or intervention* or pharmaceutical Intervention

***China National Knowledge Infrastructure***

(SU='妊娠期糖尿病'+'妊娠期高血糖'+'肥胖孕妇'+'超重孕妇'+'怀孕妇女'+'妊娠期肥胖'+'妊娠期超重'+'妊娠'+'孕妇') and (SU='益生菌'+'益生元'+'酸奶'+'乳酸菌'+'乳酸杆菌'+'微生物制剂') and (SU='血糖'+'空腹血糖'+'血糖控制'+'糖化血红蛋白'+'胰岛素敏感性'+'胰岛素疾病'+'胰岛素抵抗'+'胰岛素')

***WanFang Data***

题名或关键词: (妊娠期糖尿病 or 妊娠期高血糖or 肥胖孕妇or 超重孕妇or 妊娠期肥胖or 妊娠期超重or 怀孕妇女or 妊娠or 孕妇) and 题名或关键词:(益生菌 or 益生元 or 乳酸杆菌 or 酸奶 or 微生态制剂) and 题名或关键词:(血糖 or空腹血糖or血糖控制or糖化血红蛋白or胰岛素敏感性or胰岛素疾病or胰岛素抵抗or胰岛素)

***Chinese Scientific Journal Database***

(M=妊娠期糖尿病 OR M=妊娠期高血糖 OR M=肥胖孕妇OR M=超重孕妇OR M=妊娠期肥胖OR M=妊娠期超重OR M=怀孕妇女 OR M=孕妇 OR M=妊娠) AND (M=益生菌 OR M=益生元 OR M=乳酸菌 OR M=乳酸杆菌 OR M=酸奶 OR M=微生态制剂 OR M=微生物制剂) AND (M=血糖 OR M=空腹血糖OR M=血糖控制OR M=糖化血红蛋白OR M=胰岛素敏感性OR M=胰岛素疾病OR M=胰岛素抵抗OR M=胰岛素)

***SinoMed***

("妊娠期糖尿病"[常用字段:智能] OR "妊娠期高血糖"[常用字段:智能] OR "肥胖孕妇"[常用字段:智能] OR "超重孕妇"[常用字段:智能] OR "妊娠期肥胖"[常用字段:智能] OR "妊娠期超重"[常用字段:智能] OR "怀孕妇女"[常用字段:智能] OR "妊娠"[常用字段:智能] OR "孕妇"[常用字段:智能]) AND ("益生菌"[常用字段:智能] OR "酸奶"[常用字段:智能] OR "益生元"[常用字段:智能] OR "乳酸菌"[常用字段:智能] OR "乳酸杆菌"[常用字段:智能] OR "微生物制剂"[常用字段:智能])
